# Main Approaches to Enhance Radiosensitization in Cancer Cells by Nanoparticles: A Systematic Review

**DOI:** 10.34172/apb.2021.025

**Published:** 2020-07-13

**Authors:** Behnaz Babaye Abdollahi, Reza Malekzadeh, Fatemeh Pournaghi Azar, Fatemeh Salehnia, Ali Reza Naseri, Marjan Ghorbani, Hamed Hamishehkar, Ali Reza Farajollahi

**Affiliations:** ^1^Drug Applied Research Center, Tabriz University of Medical Sciences, Tabriz, Iran.; ^2^Department of Medical Physics, School of Medicine, Tabriz University of Medical Sciences, Tabriz, Iran.; ^3^Student Research Committee, Tabriz University of Medical Sciences, Tabriz, Iran.; ^4^Department of Operative Density, Dental and Periodontal Research Center, Tabriz University of Medical Sciences, Tabriz, Iran.; ^5^Research Center for Evidence Based Medicine, Tabriz University of Medical Sciences, Tabriz, Iran.; ^6^Imam Reza Educational Hospital, Radiotherapy Department, Tabriz University of Medical Sciences, Tabriz, Iran.; ^7^Stem Cell Research Center, Tabriz University of Medical Sciences, Tabriz, Iran.

**Keywords:** Nanoparticle, Radio-sensitization, Radiation therapy, Cancer

## Abstract

In recent years, high atomic number nanoparticles (NPs) have emerged as promising radio-enhancer agents for cancer radiation therapy due to their unique properties. Multi-disciplinary studies have demonstrated the potential of NPs-based radio-sensitizers to improve cancer therapy and tumor control at cellular and molecular levels. However, studies have shown that the dose enhancement effect of the NPs depends on the beam energy, NPs type, NPs size, NPs concentration, cell lines, and NPs delivery system. It has been believed that radiation dose enhancement of NPs is due to the three main mechanisms, but the results of some simulation studies failed to comply well with the experimental findings. Thus, this study aimed to quantitatively evaluate the physical, chemical, and biological factors of the NPs. An organized search of PubMed/Medline, Embase, ProQuest, Scopus, Cochrane and Google Scholar was performed. In total, 77 articles were thoroughly reviewed and analyzed. The studies investigated 44 different cell lines through 70 *in-vitro* and 4 *in-vivo* studies. A total of 32 different types of single or core-shell NPs in different sizes and concentrations have been used in the studies.

## Introduction

Cancer is the leading cause of mortality across most developed countries and the second main reason of death in developing countries, with more than 8.2 million deaths every year.^1-3^ Surgery, chemotherapy, and radiation therapy (RT) are three major modalities for cancer treatment.^4^ In particular, RT is one of the successful cancer treatment strategies used for more than 60% of all cancer patients.^5,6^ It causes tumor cell death by delivery of high intensity ionizing radiations to the tumor tissue.^7^

In general, the sensitivity of the highly or functionally active tumor cells is somewhat higher than that of nearby or adjacent normal tissue. Thus, the dose of radiation required to destroy cancerous tissue is far lower than that of normal tissue.^8^ However, some tumors are resistant to the radiation and their treatment require higher doses of radiation, which is out of normal tissue’s tolerance level.^8^ This limits RT application regardless of its fundamental role in cancer treatment. As a result, attempts are being made to improve the efficiency of RT mainly by: (I) enhancing the radiation dose inside the cancer cells; (II) sensitizing the tumors that are radio-resistant; (III) applying targeted RT.^9,10^ Radio-sensitizers are materials that increase sensitivity of the tumor tissue to radiation.^11^

In order to enhance radiation dose to the tumor, multiple approaches have been proposed such as metal based nanoparticles (NPs),^12,13^ quantum dots,^14,15^ super paramagnetic iron oxides,^8,16^ and non-metal-based NPs.^17^ With development of nanotechnology, NPs especially noble metal NPs, have also been developed as a hopeful approach to improve RT technique efficacy due to their unique physical and chemical properties. Radio-sensitizers have provided novel and great tools for imaging,^18,19^ diagnosing,^20^ and treating cancer.^21-24^ To date, several different NPs such as gold, iron, bismuth, titanium and carbon have been applied as probable tumor-selective radio-sensitizers.^12,25-27^ Radio-sensitizers are chemical or pharmacologic agents which enhance the response of cells to radiation. Ideal sensitizers should have these characteristics: Selectively sensitize, chemically stable and slowly metabolized, effective throughout cell cycle and effective at low daily doses of radiation. The relative efficacy of a particular cell radio-sensitizer is most often described with the sensitizer enhancement ratio (SER) or dose enhancement factor (DEF). The SER and DEF are the dose ratio of radiation alone versus in the presence of the cell sensitizer to produce the same biologic effect. If they were greater than one, then the addition of the agents is functioning as a radio- sensitizer. If they were less than one, then the drug is a radio-protector.

The mechanism of photon interactions with high-Z NPs is strongly related to radiation beam energy. However the probability of photoelectric interaction is dominant during low energy radiation, Campton scattering and pair production mainly accrue in mega voltage (MV) energies. Briefly, the basis of cellular damage with high atomic number NPs is based on generation of secondary electrons, free radicals, and reactive oxygen species (ROS).

Having explored electronic resources, it seems that no systematic review study has been done in this field. This study presents a historical report and provides a comprehensively review and analysis of the published studies. The first aim of the current study is the quantitative evaluation of the physical, chemical, and biological parameters of NPs affecting radiation enhancement. The second goal is assessing the NPs materials with a particular focus on the in-vitro outcomes as well as the principal mechanisms of response in non-metal and metal-based NPs radio sensitization. Also, we investigated the effect of the NPs type, NPs size, NPs concentration, cell line type, and radiation beam energy on the radiation dose enhancement during RT modality.

## Materials and Methods

The literature review was done and reported according to the standards set out in Preferred Reporting Items for Systematic Reviews and Meta Analyses (PRISMA) checklist.^28^

### Inclusion and exclusion criteria

Articles were included in the current review based on the following inclusion criteria: (i) the original, quantitative papers, review papers, thesis, conference papers, meetings and ongoing papers in English language; (ii) the study involved only experimental procedures, not simulation (such as Monte Carlo methods, Geant 4); (iii) studies which investigated the effect of NPs as a radio sensitizer substance in RT; (iv) photon RT not particle (proton, electron, neutron and carbon). On the other hand, articles that used NPs for drug delivery, drug formulation, shielding material or as imaging agents were excluded.

### Quality assessment

The Consolidated Standards of Reporting Trials (CONSORT) checklist^29^ was used for quality assessment of included studies. Two investigators separately rated the methodological quality of included studies.

### Search strategy, design and study selection 

A literature search was performed to find published studies that involved NPs as a radio sensitizer for cancer treatments. An organized search of PubMed/Medline, Embase, ProQuest, Scopus, Cochrane and Google Scholar was performed based on Mesh key words and suitable synonyms. Two researchers (RM and FS) independently and separately performed literature search. Our search strategy in each database was established by the following terms: ((enhancement [Title/Abstract]) AND (((“Radiotherapy”[Mesh]) OR Radiotherapy [Title/Abstract]) OR Radiation [Title/Abstract])) AND (((Metal Nanoparticles [Title/Abstract]) OR “Nanoparticles”[Mesh]). Database search had no limitation in time, and our last update on search was in March 2019. To have a comprehensive search and to find probable appropriate articles, manual search was also conducted on the reference list of articles. The search was limited to articles published in English.

### Data extraction 

**Figure 1:**
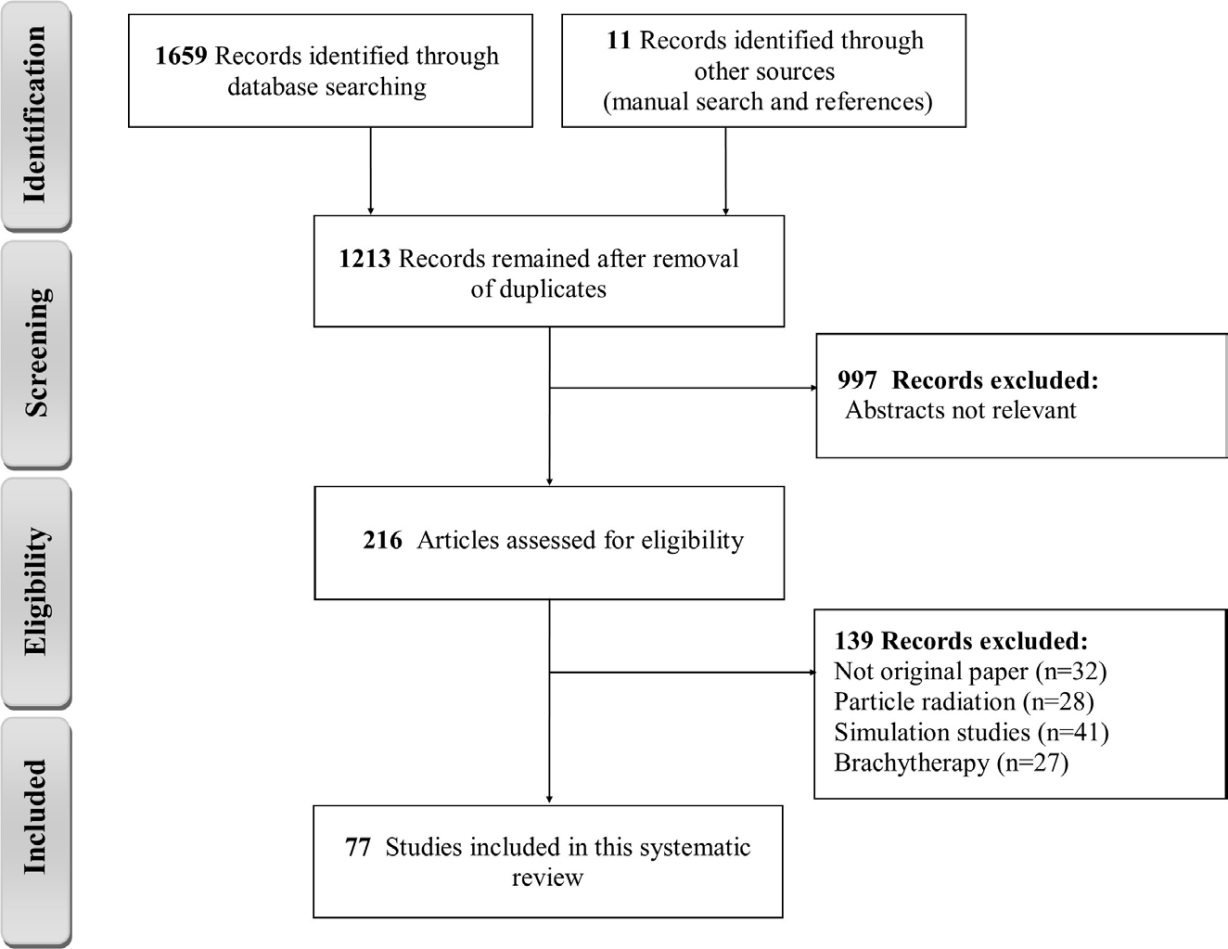
Systematic review PRISMA flow diagram.

The results of systematic literature search from the data bases were collected in Endnote X7. After removing duplicates, the articles were selected independently by two subject specialists in three stages. At first, the titles of all articles were reviewed and articles that were not consistent with the study objectives were excluded from the study. In the next step, the abstract and the full text of the articles were considered, and the full texts of relevant articles that involved inclusion criteria identified and included. For each eligible study, one reviewer extracted the data and then the results were checked by second reviewer. Any in consistencies were resolved through discussion and by consulting a third reviewer. After the final selection of studies, the required information was extracted and summarized using the extraction table. The extracted data of each study included the following content: publication year, cell line type, NPs type, NPs size, NPs concentration, photon energy, DEF or SER and mechanisms of cell damage. The total articles presented as a flow chart for the selection of the included studies (Figure 1).

## Results and Discussion

### Research results and study selection

Totally, 1670 relevant articles were identified through the literature search; of these 77 studies met the inclusions criteria which examined the effectiveness of NPs during RT and thence considered as relevant and included in the systematic review. In these studies, DEF of different NPs had been investigated in a wide range of radiation beam energy values from 88 keV to 18 MV. Specifically, 52 studies used radiation beams with less than 300 keV, 43 studies with radiation energy over 1 MV, and only six studies used Cs-137 radioactive source with 662 keV photons energy.

**Figure 2:**
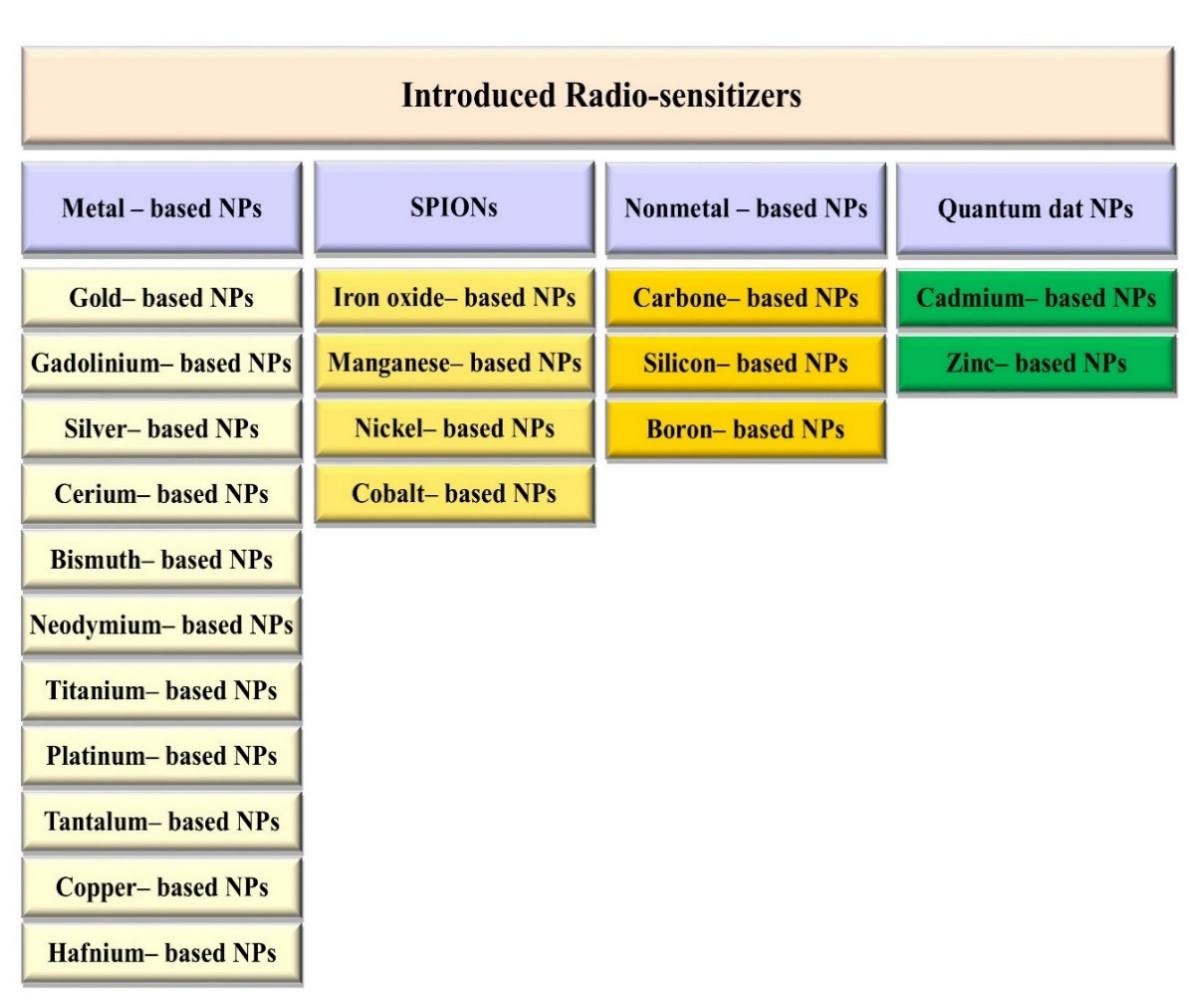
Various kinds of nanoparticles which have been introduced as radio-sensitizers in cancer radiotherapy up to date.

Various types of NPs also were used, of which the most common types were gold (Au, with atomic number 79) in 56 studies, gadolinium (Gd, with atomic number 64) in seven studies, core-shell NPs, in six studies, bismuth (Ba, with atomic number 83) in 4 studies, platinum (Pt, with atomic number 78), silver (Ag, with atomic number 47), and iron (Fe, with atomic number 26), with each used in three studies, titanium (Ti, with atomic number 22) in two studies; other NP types included hafnium (Hf, with atomic number 72), silicon (Si, with atomic number), zinc (Zn, with atomic number 30), neodymium (Nd, with atomic number 60), lanthanide (La, with atomic number 57), cerium (Ce, with atomic number 58), tantalum (Ta, with atomic number 73), which each used in one study (Figure 2).

### Principal finding

NPs have received special attention over the last decade and they have been studied as one of the best ways to improve radiation dose enhancement agent. Significant studies have been conducted to determine the NPs’ radio sensitization effect. The main finding of the literature review was that NPs considerably enhanced the radiation dose in loaded tissues. All studies recommended the use of NPs to enhance the effects of radiation at the tumor site. The results indicated a DEF of 1.01 to 2.95 (1.44 ± 0.43) depending on the NPs type, NPs concentration, NPs size, cell line, radiation energy, and delivery condition. Table S1^30-106^ (See online Supplementary file 1) summarizes the main results of the involved studies such as the NPs’ size, NPs’ concentration, cell line types, and radiation energy.The sensitizing characteristics of NPs have been established on various cell lines. To better compare the effect of different NPs concentrations, the reported concentrations in the articles were converted to the same unit (mM). Different NP sizes, concentrations, cell lines, radiation energies, and doses had been used in the included studies. Among NPs, Au NPs were the most studied NPs in cancer therapeutics.

### Mechanisms of radio-sensitization by NPs at biological systems

The integration of nanotechnology with biotechnology allows us to deliver high atomic number metal NPs to the target cells as radio-sensitizers due to their elevated absorbance properties in comparison to surrounding soft tissue.^107^ Further, with increasing experimental and simulation studies in the field, it was found that the DEF values obtained in experimentally biological *(in-vitro* and *in-vivo*) studies were considerably greater than those found by the simulation (mass attenuation coefficients) studies in both kilo-voltage and mega-voltage radiation energy. These findings reported other mechanisms through which NPs sensitize cells to radiation. Indeed, based on the results of previous studies, it can be concluded that the mechanism of NPs radiation dose enhancement in biological systems is classified into three sections: 1) physical phase, 2) chemical phase, and 3) biological phase.^108,109^ Thus, the disagreement between DEF values of experimental and simulation studies can be explained by the lack of chemical and biological phases on cellular damage in simulation studies.

The physical phase of radio-sensitizers is based on the photoelectric effect, coherent and incoherent scattering, and pair production. Each photon can interact with the matter and be completely absorbed or be scattered and thus deposit part or all of its energy according to three possible mechanisms.^110^ The probability of each three different mechanism depends on the photon energy and the atomic number of the radio sensitizer. Further, original studies suggest the key NPs chemical characteristics including size, shape, crystal structure, surface area, surface energy, surface characteristics, and inner structure generally influence the toxic manifestations of these materials.^111,112^

The biological effects of NPs are a major cause for cell death. They reflected in cells as increased ROS and oxidative products, as well as elimination of cellular antioxidants. Thus, according to studies to date, three important biological pathways for radio sensitization have been introduced: (1) oxidative stress,^113^ (2) cell cycle disruption, and (3) DNA repair inhibition.^109,114^

### NPs Size, surface area, and chemistry

NPs surface chemistry is a key parameter which affects bio-distribution and cellular uptake of NPs. NPs are single elements with a diameter of 1 to 100 nm. The smaller the NP diameter, the larger the surface area/volume ratio. The larger surface area causes more atoms to form around the surface of NPs, making NPs highly reactive and conferring them new and unique physicochemical properties. Thus, an important difference between NPs and micro-particles is their surface area. NPs are capable of interacting radiation due to their larger surface area thereby enhancing the physical, chemical, and biological effects.^115,116^ Also, the smaller the NP size, the longer they remain in the blood circulation. Smaller NPs are filtrated through kidneys quickly, while larger ones avoid clearing.

There are studies proving that NPs of any compound are more cytotoxic than MPs of the same compound.^117,118^ In the study by Gurr et al, they disclosed a strong relationship between the size and toxicity of TiO_2_ NPs. TiO_2_ NPs with diameter < 50 nm caused increased micronuclei number in human bronchial epithelial cells, while TiO_2_ micro particles with diameter > 200 nm were nearly harmless at the same concentration.^119^

As cellular uptake of the NPs depends on surface chemistry, Chompoosor et al determined the effect of Au NPs surface functionality on ROS generation and DNA damage. They approved that the cytotoxicity and genotoxicity of Au NPs depend on their surface chemistry (hydrophobicity). Increasing the hydrophobicity of the particles improved their cytotoxicity.^120^ Also, the majority of studies have found that size is an influential radiation sensitivity parameter. Large-sized Au NPs have the most efficient DEF. Wang et al^50^ examined the *in-vitro* Au NPs radiosensitization effect in the breast cancer cell line (MDA-MB-231). DEFs of 1.49 and 1.86 were observed with 16 nm and 49 nm Au NPs, respectively. Brun et al^56^ prepared a comprehensive study for size effect of Au NPs for radiosensitization. They found that DEFs of 1, 1.75, 1.76, 2.65, and 2.95 were obtained associated with 8 nm, 20 nm, 37 nm, 74 nm and 92 nm of Au sample size, respectively. Similarly, Bobyk et al^32^ showed that use of 1.9 nm and 15 nm caused DEF 1.92 and 1.40, respectively. Also, a systematic study of the size-dependent radiosensitization of Au NPs against HeLa cell line found that 4.8 nm, 12.1 nm, 27.3 nm, and 46.6 nm Au NPs revealed DEFs of 1.41, 1.65, 1.58, and 1.42 obtained at the same concentration and radiation energy, respectively. Since only NPs with size 1-100 nm are able to enter cells, optimal size design can enhance the cell internalization and consequently results.

### NPs’ type and concentration

Based on literature review, the effect of NPs’ concentrations on dose enhancement is far greater than NPs’ size. Elevation of the NPs’ concentration reduces the cell growth rate. This decrease seems reasonable as the concentration rise of NPs increases the number of NP atoms. Thus, more physical, chemical, and biological interactions occur between cells, photons, and atoms. Higher NP concentrations seem to carry a higher risk of toxicity. Thus, the balance between dose enhancement effect and toxicity should be set. Butterworth et al^33^ examined the radiation enhancement of Au NPs in several cell lines with two different concentrations at 160 kVp photon irradiation. The following DEFs have been reported for various cell lines using 0.05 and 0.5 mM concentrations of Au NPs: DEFs of 0.86 and 0.87 (L 132), 1.04 and 0.96 (Astro), 1.16 and 1.97 (AGO), 1.30 and 1.91 (T98G), 1.67 and 1.11 (MDA 231), 1.41 and 1.09 (MCF7), 1.07 and 1.02 (PC3), and 0.98 and 0.81 (DU145), respectively. In another study, Rahman et al^47^ examined the effect of different concentrations of Au NPs on enhancing the radiation effects. They revealed that DEFs significantly increased using a high concentration of NPs. DEFs of 2.4 and 2.0 were noted while using 1mM and 0.5 mM at 80 kVp, respectively. Also, DEFs of 2.2 and 1.4 were obtained using 1mM and 0.5 mM at 150 kVp, respectively.

Ahamed et al explored the effects of TiO_2_ and Pb NPs toxicity in human lung epithelial (A549) cells. They found that TiO_2_ was not toxic to A549 cells. However, cell viability diminished due to Pb-induced toxicity, production of ROSs, and reduction in antioxidant levels. Interestingly, in co-exposure group (TiO_2_  NPs + Pb), TiO2 significantly reduced Pb toxicity in A549 cells. Cellular uptake confirmed that TiO_2_ NPs increased the bioaccumulation of Pb in cells.^121^

Several studies confirmed that Ag NPs mostly cause significant cytotoxicity.^122^ As for Pt, the Pt NPs were shown to enter the cells through diffusion, leading to an increase in DNA damage and apoptosis.^123^ Asharani et al compared the toxicity between 3–10 nm Pt, 5–35 nm Ag, and 15–35 nm Au NPs covered with PVA, and concluded that Ag NPs were the most toxic, followed by Pt NPs, while Au NPs presented no indication of toxicity.^124^

### Radiation energy

The choice of optimal beam energy in using a dose-enhancing agent is an important consideration. Several reports have shown the efficiency of NPs’ radio-sensitization at low energy beam (kV). Meanwhile, such radio enhancement is shown at MV X-rays. Initial RT was carried out with kV energy range. With advances in RT and the potential damage of low-energy radiation to the skin, today, most RT units use clinical linear accelerators for producing MV X-rays.

**Figure 3:**
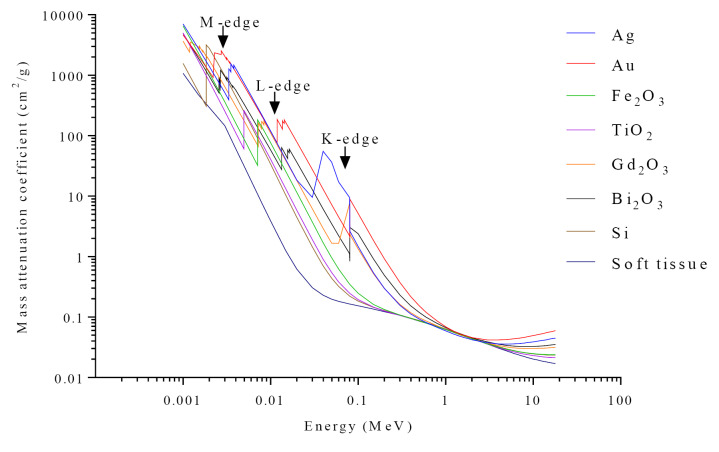
Mass attenuation coefficients of suggested nanoparticles and soft tissue versus different radiation with 1keV until to 18 MeV energies. Plot data obtained from WinXCom software.

In order to find the best radiation energy used in RT to produce the most effective dose enhancement effect in a tumor, we need to have a closer look to the interactions between radiation and matter. Theoretical principles of X-ray interactions with NPs have already been described.^125^ The interaction of photons with materials at low energies is based on the photoelectric phenomenon. The photoelectric effect exhibits a cross-section with Z^3^/E^3^ depending on the photon energy and material atomic number, and is enhanced due to increased absorption by electron shells (K, L, M, etc.) as shown in Figure 3. Thus, when only a small amount of NPs with a high atomic number is delivered to the tumor, the photoelectric cross-section significantly grows and absorbs considerably more energy per unit of mass than the soft tissue, which is typically 10 to 150 times greater than surrounding soft tissue at low energies. With the increase in the photon energy in RT, Compton scattering and Pair production (photon energies >1.022 MeV) occur with a higher probability than photoelectric effect. Note that the radiation beam of the linear accelerators has a spectrum of energy (poly-energetic), so the low-energy component of the spectrum releases energy by photoelectric effect where high-energy components are more likely to interact by Compton effect and pair production. On the other hand, Compton scattering does not depend on the Z of the materials; Pair production is also a function of Z^2^, so the relative effect of NPs for Au NPs to soft tissue is approximately 127 (79^2^/7^2^).

As a result of all these mechanisms, secondary electrons (nearly 10^4^ per MeV) are produced at the tumor site, which has a very low energy (< 50 eV) and low linear energy transfer (LET).^126^ Thus, their energy is left to their immediate surroundings and damaging target cells. It is well known that secondary electrons induce significant single- and double-stranded breaks in DNA due to the rapid collapse of molecular resonance located in the DNAs basic components (i.e., the bases, sugar and phosphate group analogs and oligonucleotides) and of proteins (e.g. amino acids).^127-129^

The mass attenuation coefficients of applied NPs and soft tissue are shown in Figure 3. These attenuation curves show that NPs are considerably more absorbent, especially around certain energies that are related to the K-edge, L-edge, and M-edge.^130^ As expected, DEF depends on radiation energy. According to review results, such following DEF for variation radiations have been described: DEFs of 2.9 and 3.7 using 0.5 mM, a concentration of 1.9 nm Au NPs at 6 MeV and 12 MeV were reported. DEFs of 1.66, 1.43 and 1.17 were observed with 105 kVp, 220 kVp and 6MV X-rays, respectively. DEFs of 1.44, 1.1 and 1.32 were achieved with 8 keV, 160 kVp and 6 MV X-rays, respectively. DEFs of 2.0-3.7 and 1.8-3.0 were reported while using 90 keV and 50 keV, respectively (for different sizes and concentrations).

It is expected that the radio-sensitization of NPs is insignificant at MV energies due to the negligible contribution of photoelectric absorption of photons. Chithrani et al assessed the dependence of radio-sensitization at the clinically relevant radiation energy. They reported a decrease in radio-sensitization (DEFs were 1.66 at 105 kVp, 1.43 at 220 kVp, 1.18 with 660 keV) with increasing energies. Similarly, Jain et al^41^ observed that DEFs of 1.41, 1.29 and 1.16 were acquired in MDA-MB-231 cells by 1.9 nm Au NPs in combination with 160 kVp, 6 MV, and 15 MV X-rays, respectively. To sum up, in almost all studies, the dose enhancement of photons with MV energies is lower than kV energies, but still far greater than the MC simulation, which is due to the biological effect of NPs.

### Cell line type effect

The cytotoxicity of NPs varies in different cell types. NPs could enhance the sensitivity of some cells to irradiation but not all cells, as glucose-coated Au NPs did not radio sensitize human diploid fibroblast cells but did enhance human prostate carcinoma cells. As another proof, despite cellular uptake in human prostate cancer cells and lung epithelial cells, radiosensitization was observed in neither of them. Au NPs’ cellular uptake levels and cell cycle phases might justify it. Metallic materials block cells at the G2/M phase, the most radiosensitive phase of the cell cycle, and therefore augment cell radio-sensitivity.

Albanese and Chan investigated the effect of Au NPs aggregation with different sizes on cellular uptake and toxicity in three different cell lines. Their contrasting results suggested that cell type may play a significant role. It was found that, while there was no difference in the toxic response of single and aggregated NPs, the uptake patterns had a clear difference between single and aggregated NPs. There was a 25% reduction in the uptake of aggregated NPs with HeLa and A549 cells compared to single NPs. However, there was an increase of 200% in cell uptake of MDA-MB 435 for the largest synthesized aggregates.^131^ Similarly, Jain et al^40^ evaluated the cytotoxicity of 1.9 nm Au NPs in normal L132, prostate cancer DU145, and breast cancer MDA-MB-231 cells in combination with 6 megavoltage X-ray. DEFs of 1.08, 1.13 and 1.29 were achieved in L132, Du 145 and MDA-MB-231 cells, respectively. Due to the same NP and radiation energy, this cannot be contributed to levels of radiosensitization based on physical action. So it obviously showed that different cell lines had a various biological response to Au NPs. Au NP chemo-sensitization was observed in MDA-MB-231 cells treated with bleomycin which approved different biological respond. Similar results were shown in McMahon et al study.^132^ This suggests that some cell lines show little or no radio-sensitization despite taking up similar numbers of NPs.

### Generation of ROS

Although the main mechanism whereby NPs induce cell damage effects is still unknown, it has been suggested by different biological studies that they can produce ROS, and therefore can affect the concentration of intracellular calcium, activate transcription factors, and induce cytokine production.^133,134^ ROS can damage cancer cells in several ways, such as DNA damage, interfering with signaling functions, and modulating gene transcription.^135^ The most commonly produced ROS in biological systems include anion superoxide (O_2_), hydrogen peroxide (H_2_O_2_,), and hydroxyl reactive radicals (-OH). The extent of damage caused by ROS depends not only on the type and amount of ROS, but also on the time and duration of exposure to ROS and the external factors of the cell, such as temperature, oxygen pressure, and the surrounding environment including ions and proteins.^136^ In physiological conditions, the concentration of H_2_O_2_ is low and about 5-50 nM. If the H_2_O_2_ concentration reaches 1-3 μM, apoptosis (planned cell death) induction happens. For concentrations above 3 μM, it becomes toxic to the cell and leads to its necrosis (un-programmed cell death pathway).^137,138^ Antunes and Cadenas verified that by increasing certain ROSs (e.g., H_2_O_2_), the cell’s viability declines.^139^ Shukla et al assessed genotoxicity of TiO_2_ NPs on human epidermal cells (A431) as an *in vitro* model.^140^ They observed a statistically significant relationship between ROS generation and DNA damage and micronucleus formation on exposure to TiO_2_ NPs group. Their results are in agreement with Kang et al. who reported that TiO_2_ NPs induced ROS generation in human lymphocytes.^141^ Also, they have been described cell–specific DNA DSB formation, cytokinesis arrest, and apoptosis in the absence of radiation with 30 nm Au NPs.

Nonmetal NPs (silicon and carbon-based NPs) can also induce radiosensitization effects based on an oxidative stress mechanism. Si NPs significantly improved ROS production in glioma C6 and MCF-7 cells under X-ray irradiation. Positively charged NH_2_-Si NPs penetrated the mitochondrial membrane and significantly raised intracellular ROS levels in MCF-7 cells under radiation.^103^

**Figure 4:**
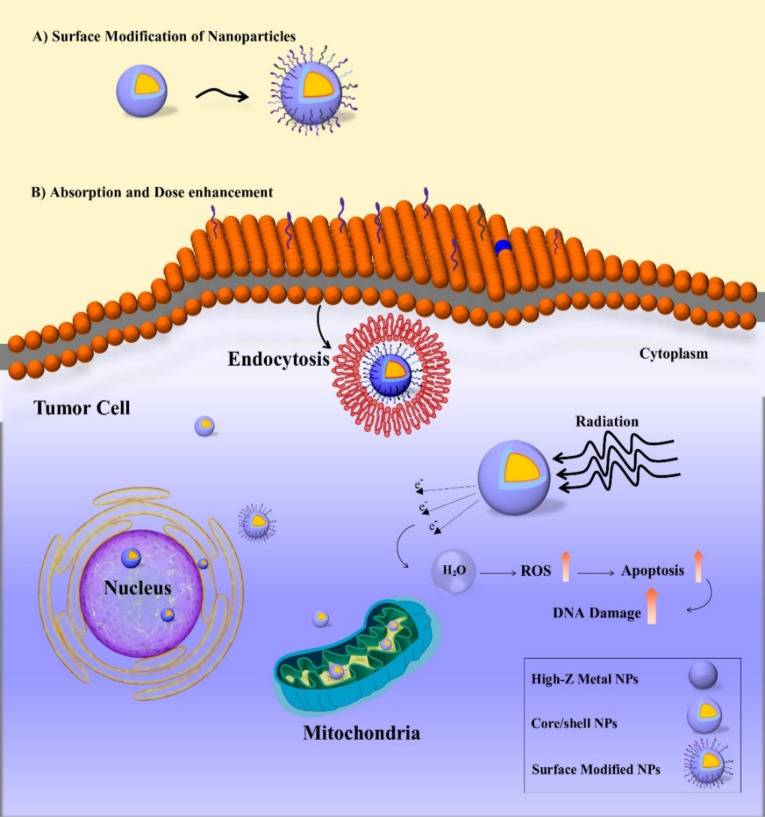
Enhanced radio-sensitivity of cancer cells by nanoparticles with mitochondria and nucleus targeting.

ROS generation has also been proposed as a possible mechanism by ionizing radiation.^142^ Geng et al^39^ showed that Au NPs enhanced the production of intracellular ROS when irradiated with 90 kVp or 6 MV X-rays in SKOV-3 human ovarian cancer cells. Wason et al investigated whether and to what extent cerium oxide NPs might affect radiation-induced ROS production in pancreatic cancer cells and normal pancreatic cells.^143^ Surprisingly, the results disclosed a 200% increase in radiation-induced ROS generation in L3.6pl cells with cerium oxide NPs compared to cells exposed to radiation alone. These results indicate that increasing ROS production by NPs may be one of the mechanisms that facilitates NPs radio sensitivity. The enhanced radio-sensitivity of cancer cell by NP is shown in Figure 4.

Definitely, NPs supported RT open up new perspectives in the fight against cancer. However, there are some problems and limitations that prevent it from translated into the clinic. It was found that the promising preclinical results of *in vitro* studies did not fully match with the *in vivo* experiments. This may be due to disparity between predicted levels of radiosensitization based on physical and biological actions. Also, reviewing the studies, showed that the radiosensitization of a particular cell line depends on many factors, and even different cell lines show different responses to the NPs. So to boost efficiency, specific NPs must be used for each cell line. Moreover, interesting is the existence of various mechanisms of cell damage by NPs. Simultaneous use of several types of NPs or the use of Nano-complexes to increase the efficiency of treatment as a radio-sensitizer can be very promising in the future.

## Conclusion and future perspectives

The studies conducted on the potential of using NPs in RT have confirmed the role of these various NPs in enhancing the radiation dose in loaded tissues. NPs have proved to cause radio-sensitization at kilo-voltage and megavoltage photon energies. RT dose enhancement with NPs appears to be a promising approach for improved cancer treatment. Different NPs sensitize cancer cells to RT through multiple mechanisms, including oxidative stress, DNA damage, cell-cycle arrest and apoptosis. For successful RT, it seems to use, (1) NPs with high atomic number (Z) to enhance RT efficacy via their photoelectric and Compton effects, (2) targeting cancer cells with specific targeting molecules extends the circulation time of the NPs to increase their accumulation in cancer cells and (3) the combination of two different types of radio-sensitizers or the combination of radio-sensitizers can result in significantly synergistic tumoricidal effects. Finally, using NPs can be an asset not just to radio-sensitize cells but also to provide contrast as they can be imaged, which can lead to better drug tracking and detection of the exact location of the tumor for RT. Despite their unique merits, it is difficult to move toward clinical programs without understanding the mediating mechanisms of biological effects in cells.

## Ethical Issues

Not applicable.

## Conflict of Interest

The author declares that there is no conflict of interest.

## Acknowledgments

This study was supported by Research Center for Evidence Based Medicine (RCEBM), Tabriz University of Medical Sciences, Tabriz, Iran.The authors would like to thank Director of Research Development and Coordination Center (RDCC), Dr. Ghojazadeh, for his assistance withresearch methodology.

## Supplementary Materials

Supplementary file 1 contains Table S1
