# Supplementary material for: Main Approaches to Enhance Radiosensitization in Cancer Cells by Nanoparticles: A Systematic Review
Source: Adv Pharm Bull. 2020 Jul 13;11(2):212–23. doi: 10.34172/apb.2021.025 (PMC8046397; doi:10.34172/apb.2021.025)
Supplement: Supplementary file 1 — contains Table S1 [file apb-11-212-s001.pdf]

Supplementary file 1

Table S1. A summary of the results and main factors on the radiation dose enhancement in each study

Ref.	Author Name	Year	Country	SD	NPs	Size (nm)	Con	Dose	Exposed	Cell line	Mechanism	DEF or SER
30	Chien et al	2007	-	In vitro	Au	20	1 mM	-	6 MeV	CT-26	-	1.19
31	Liu et al	2010	Taiwan	In vitro	Au	6.1	0.5 mM	-	6.5 keV	CT-26	Apoptosis	2
									160 kVp			1.1
									6 MeV			1
32	Bobyk et al	2013	France	In vitro	Au	1.9	50 mM	6 Gy	50 keV	F98 glioma	DNA damage	1.92
						15						1.40
						1.9 and 15	76 mM	15 Gy	88 keV	F98 glioma		-
33	Butterworth et al	2010	UK	In vitro	Au	1.9	50 and 500 mM	-	160 kVp	L 132	ROS and DNA damage	0.86 and 0.87
										Astro		1.04 and 0.96
										AGO		1.16 and 1.97
										T98G		1.30 and 1.91
										MDA 231		1.67 and 1.11
										MCF7		1.41 and 1.09
										PC3		1.07 and 1.02
										DU145		0.98 and 0.81
34	Chattopadhyay et al	2013	Canada	In vitro	Au	30	12 mM	11 Gy	100 kVp	MDA-MB-361	DNA damage	1.6 (targeted)
												1.3 (non-targeted)
35	Chen et al	2015	China	In vitro	Au	18	182 mM	-	160 kVp	U87	Apoptosis	1.37
				In vivo (mice)		18	6.5 mM			U87		-
36	Chithrani et al	2010	Canada	In vitro	Au	50	1mM	-	150 kVp	HeLa	DNA	1.66

Supplementary file 1

									220 kVp			1.43
									660 keV			1.18
									6 MV			1.17
37	Coulter et al	2012	UK	In vitro	Au	1.9	0.012 mM	3 Gy	160 kVp	DU14, MDA-MB-23, L132	ROS and cell cycle arrest	-
38	Cui et al	2014	Canada	In vitro	Au	2.7	2.53 mM	-	225 kVp	MDA-MB-231	Apoptosis	1.04 -1.44 related to different incubation time
39	Geng et al	2011	China	In vitro	Au	14.37	-	-	90 kVp and 6 MV	SK-OV-3	ROS	-
40	Jain et al	2014	UK	In vitro	Au	1.9	0.012 mM	-	160 kVp	MDA-MB-231	-	1.01 -1.41 related to different oxygen concentrations
41	Jain et al	2011	UK	In vitro	Au	1.9	0.012 mM	-	160 kVp	L132	DNA damage	1.05
									6 MeV			1.08
									160 kVp	DU145		0.92
									6 MeV			1.13
									160 kVp	MDA-MB-231		1.41
									6 MeV			1.29
									15 MeV			1.16
42	Joh et al	2013	USA	In vitro	Au	12	1 mM	4 Gy	150 kVp	U251	DNA damage	1.3
43	Kaur et al	2013	India	In vitro	Au	5-15	0.027 mM	-	Co-60	HeLa	-	1.52
									62 MeV			1.39
44	Khoshgard et al	2014	Iran	In vitro	Au	50	0.050 mM	-	120 kVp	HeLa	Apoptosis and ROS	1.40
									180 kVp			1.43

Supplementary file 1

									200 kVp			1.39
									250 kVp			1.39
									Co-60			1.03
45	Kong et al	2008	Canada	In vitro	Au	10.8	0.015 mM	-	220 kVp	MCF-7	-	1.3
46	Liu et al	2008	Taiwan	In vitro	Au	4.7	0.5 mM	-	6 MeV	CT26	Cell cycle arrest	-
47	Rahman et al	2009	Australia	In vitro	Au	1.9	1 mM	-	80 kVp	BAEC	-	2.46
									150 kVp			2.2
					Au	1.9	0.5 mM		80 kVp	BAEC		2.0
									150 kVp			1.4
48	Roa et al	2009	Canada	In vitro	Au	10.8	15 nM	-	662 keV	DU-145	Cell cycle arrest	1.22-1.37 after treating cells for 2 and 6 h.
49	Taggart et al	2014	Ireland	In vitro	Au	1.9	2538 mM	2 Gy	225 kVp	MDA-MB-231	DNA damage	1.23
								4 Gy				1.20
								8 Gy				1.17
								2 Gy		DU-145		1.01
								4 Gy				1.06
								8 Gy				1.1
								2 Gy		T98G		1.90
								4 Gy				1.57
								8 Gy				1.35
50	Wang et al	2013	China	In vitro	Au	13	20 nM	-	6 MeV	A549	Apoptosis	1.49
51	Wang et al	2015	China	In vitro	Au	16	20 nM	-	6 MeV	MDA-MB-231	ROS	1.49
						49						1.86

Supplementary file 1

52	Zhang et al	2008	Canada	In vitro	Au	30	15 nM	-	200 kVp	DU-145	Apoptosis	1.5-2
53	Zhang et al	2012	Canada	In vitro	Au	4.8	0.05 mM	-	662 keV	HeLa	Apoptosis	1.41
						12.1						1.65
						27.3						1.58
						46.6						1.42
54	Zhang et al	2014	Canada	In vitro	Au	2	253 mM	-	662 keV	HeLa	DNA damage	1.3
55	Maggiorella et al	2016	France	In vitro	Hf	50	-	-	6 MeV	HT1080	-	1.80
56	Brun et al	2009	France	In vitro	Au	8	5 nM	-	49 keV	DNA	DNA damage	1
						20						1.75
						37						1.76
						74						2.65
						92						2.95
57	Yang et al	2008	USA	In vitro	CdSi@ ZnS	2.1	-	-	6 MeV	H460	-	0.631
58	Štefančíková et al	2014	France	In vitro	*GdBN*	3	0.5 mM	-	1.25 MeV	U87	-	0.23
59	Zhao et al	2012	China	In vitro	Fe_3O_4@Ag	30	50 µg/L	0, 1, 2, 4, 6, and 8 Gy	-	CNE	Apoptosis	2.262
60	Yi et al	2016	China	In vitro	*Au@MnO*$_2$	50	20 µg/mL	6 Gy	160 keV	4T1	ROS and DNA damage	Significant DNA damage
61	Rezaee et al	2017	Iran	In vitro	*Au*	99	0.1 mM	-	6 MV	HT-29	Apoptosis	1.17
										CHO		1.15

Supplementary file 1

62	Gong et al	2018	China	In vitro	TaOx@MnO_2	100	25 µg/mL	-	6 Gy	4T1	ROS	Remarkably promote the efficacy of RT
63	Darfarin et al	2018	Iran	In vitro	AuN@SiO_2	23	200 mg/mL	2, 4, 8 Gy	6 and 18 MV	MCF-7	Cell cycle and apoptosis	-
					AuS@SiO_2	31						
64	Zabihzadeh et al	2018	Iran	In vitro	Au	24	-	-	18 MV	HT-29	DNA damage	1.25
65	Ahn et al	2017	Korea	In vitro	Au	-	10 mM	2 Gy	-	PC-3	-	SER was 1.21
66	Alqathami et al	2016	USA	-	Bi_2O_3	50	0.5 mM	-	100 kV	phantom	-	1.90
					Au							1.77
					Bi_2S_3	5	0.25 mM		150 kV			1.38
					Au							1.51
					Bi_2O_3	50	0.1 mM		100 kV			1.52
					Au							1.45
67	Antosh et al	2015	USA	In vitro	Au	1.4	-	-	250 kVp	A549	DNA damage	-
68	Banaee et al	2016	Iran	-	ZnO	-	6150 mM	10 Gy	6 MV	phantom	-	1.57
							12300 mM					1.69
							36900 mM					1.78
							49200 mM					1.82
69	Baronia et al	2018	India	In vitro	Au	2-4	-	2 Gy	-	PC3	-	-
70	Behrouzki et al	2019	Iran	In vitro	Au	30	2.53 mM	-	6 MV	Gel	-	1.1
						50						1.17

Supplementary file 1

						100						1.12
71	Choi et al	2018	Korea	In vivo (12 BALB/C mice)	Au	20	0.039 mM	0-6 Gy	-	MDAMB-231	ROS	-
72	Cruje et al	2015	Ca	In vitro	Au	14	-	-	220 kVp	HeLa and MDA-MB-231	-	-
73	Cui et al	2017	Ca	In vitro	Au	6	2.53 mM	-	225 kV	MDA-MB-231	Apoptosis	1.14
74	Delrme et al	2017	Fr	In vitro	Gd	3	13.37 mM	4 Gy	1.25 MV (Co 60)	F98	DNA damage	1.2
75	Engels et al	2016	Australia	In vitro	Ta_2O_5	-	0.113 - 1.13 mM	-	90 keV	9L	DNA damage	-
76	Farahani et al	2017	Iran	-	Bi_2O_3	40	0.1 mM	-	Co-60	Gel	Apoptosis	30% enhancement
					Gd_2O_3							21% enhancement
77	Hainfeld et al	2004	USA	In vivo (Balb/C mice)	Au	2	-	-	250 kVp	Balb/C	-	-
78	Hauser et al	2016	USA	In vitro	Fe_2O_3	9-12	626 mM	5 Gy	-	A549	ROS	-
79	Huang et al	2015	USA	In vitro	Au	50 nm	0.140 mM	4 Gy	6 MV	MDA-MB-231	DNA damage	1.1
								2 Gy	225 kVp			1.5
80	Zhang et al	2018	China	In vitro	Au	6.3±1.1	400 nM	6 Gy	6 MV	LS180	Apoptosis	-
81	Yu et al	2017	China	In vitro	Ag	20	-	2, 4, 6 and 8 Gy	6 MV	CNE	Apoptosis and Cell cycle arrest	-
82	Youkhana et al	2017	Australia	In vitro	TiO_2	30 ± 5	0.5, 1 and 4 mM	-	80 kVp	HaCaT	ROS	1.17, 1.34 and 1.56
									6 MV			1.13, 1.18 and 1.37
									80 kV	DU145		1.21, 1.4 and 1.68

Supplementary file 1

									6 MV			1.1, 1.19 and 1.43
83	Yoo et al,	2016	Ca	In vitro	$Gd(OH)_3$	-	0.144 and 0.360 mM	3 Gy	300 kV	MDA-MB-231	-	-
84	Karim et al.	2016	USA	In vitro	Au	1.4	5 mM	2 Gy	220 kVp	PC-3	-	2.65
85	Xu et al	2012	China	In vitro	Au	15	0.253 mM	2 Gy	6 MV	A375	Cell cycle arrest	1.14
86	Wu et al	2016	China	In vitro	Au	15.38 ± 4.13	0.1 mM	2 Gy	6 MV	U251	ROS	-
87	Wagner et al	2016	CA	In vitro	Pt	1-2	0-5.1 mM	2 Gy	125 kVp	BT-474, MDA-231, BT-549 and MCF-7	-	-
88	Taupin et al	2015	FR	In vitro	Gd	3.0 ± 1.0	13.37 mM	-	25 to 80 keV and Co-60	F98	Apoptosis	-
89	Swanner et al	2015	USA	In vitro	Ag	23 ± 14	0 – 934 mM	0-4 Gy	300 kV	184B5, MCF-7, MCF-10A, MDA-MB-231, HMEC, BT-549, and SUM-159	DNA damage and ROS	-
90	Shi et al	2016	CA	In vitro	Au	2.77±0.69	1.27 mM	1–10 Gy	26 keV	HCT116	Cell cycle arrest	SER of 1.69
							0.5 mM	1–10 Gy				SER of 1.48
91	Saberi et al	2016	Iran	In vitro	Au	50	0.080 mM	2 Gy	9 MV	HT-29	Apoptosis	SER of 1.4
92	Rashid et al	2018	Malaysia	In vitro	Au	1.9 nm	1 mM	-	6 and 10 MV	HeLa	Apoptosis	-

Supplementary file 1

93	Popovtzer et al	2016	Israel	In vivo (36 mice)	Au	30	-	-	6 MV	A431	Apoptosis	-
94	Pagácová 1 et al	2019	Czech Republic	In vitro	Au	2.4	0.5 mM	2 and 4 Gy	Cs-137	HeLa, U87, and SKBr-3	DNA damage	-
					Pt	2.6	0.5 mM	2 and 4 Gy	Cs-137	HeLa, U87, and SKBr-3		-
95	Morita et al	2016	Japan	-	PAA-TiO_2/H_2O	124 ± 65	5.0 mg/ml	-	X-rays	distilled water	ROS	-
96	Mafakhei et al	2016	Iran	In vitro	Fe_2O_3	-	0.125 mM	-	6 MV	HeLa	Apoptosis	1.19±0.15
							0.313 mM					1.49±0.11
97	Luchette et al	2014	USA	In vitro	Gd	5	0.5 mM	4 Gy	220 kVp	HeLa	Apoptosis	SER was 1.54
								4 Gy	6 MV			SER was 1.28
98	Lu et al	2019	UK	In vitro	La_2O_3	100	0.040 mM	0 and 3 Gy	250 kV	U-87 MG and Mo59K	ROS	-
					CeO_2	50						
					CeO_2-Gd	100						
					Nd_2O_3	100						
					Gd_2O_3	100						
99	Liu et al	2010	Taiwan	In vitro	Au	10	0.500 mM	-	6 MeV	EMT-6	Cell cycle arrest	-
100	Liu et al	2018	China	In vitro	Ag	26.87 ± 3.68	0.299 mM	-	6 MeV	U251	Cell cycle arrest	SER was 1.78
							0.324 mM					SER was 1.34
							0.255 mM			C6		SER was 1.84
							0.303 mM					SER was 1.45
101	Berbeco et al	2012	USA	In vitro	Au	50	0.25 mM	4 Gy	6 MV	HeLa	DNA damage	-
102	Cui et al	2014	China	In vitro	DOC and DOC-NPs	85	-	-	4 MeV	BGC823	ROS and	SER were 1.09 and 1.35

Supplementary file 1

										SGC7901	cell cycle arrest	SER were 1.02 and 1.2
										MKN45		SER were 1.11 and 1.21
										GES-1		SERs were 1.05 and 1.07
103	Gara et al	2012	Argentina	In vitro	Si	3±1	17.8 mM	-	4 MeV	C6	ROS	-
104	Jin et al	2007	China	In vitro	paclitaxel-loaded PLGA	500	-	-	1.25 MeV	MCF-7	ROS and cell cycle arrest	SER was 1.4
105	K.A et al	2018	Malaysia	In vitro	Pt	29	0.1 mM	-	6 MV	HeLa	Apoptosis	SER was 1.96
						36						SER was 2.31
						42						SER was 2.27
						52						SER was 1.77
106	Khoie et al	2014	Iran	In vitro	Fe_3O_4	20	4.3 mM	2 Gy	6 MV	DU145	DNA damage	1.24
								4 Gy				1.22
								6 Gy				1.21

SD: Study design, NPs: Nanoparticles, Con: concentration, DEF: dose enhancement factor and SER: Sensitizer enhancement ratio,PAA-TiO_2/H_2O: Poly acrylic acid-modified titanium dioxide with hydrogen peroxide, DOC: Docetaxel and Docetaxel-loaded-poly (ethyleneglycol)-poly (varepsilon-caprolactone) NPs , PLGA: poly(D,L-lactide-co-glycolide) NPs containing paclitaxel, ROS: reactive oxygen species.
